# Comparing ART outcomes in women with endometriosis after GnRH agonist *versus* GnRH antagonist ovarian stimulation: a systematic review

**DOI:** 10.1177/20420188231173325

**Published:** 2023-07-04

**Authors:** Kevin K.W. Kuan, Sean Omoseni, Javier A. Tello

**Affiliations:** School of Medicine, University of St Andrews, St Andrews, UK; Edinburgh Medical School, University of Edinburgh, Edinburgh, UK; School of Medicine, University of St Andrews, St Andrews, UK; School of Medicine, University of St Andrews, St Andrews KY16 9TF, UK; Biomedical Sciences Research Complex, University of St Andrews, St Andrews, UK; Centre for Biophotonics, University of St Andrews, St Andrews, UK

**Keywords:** assisted reproductive technology, endometriosis, GnRH agonist, GnRH antagonist, infertility, ovarian stimulation

## Abstract

**Background:**

Endometriosis is an oestrogen-dependent disease that can cause subfertility in women who may require assisted reproductive technology (ART) to achieve their pregnancy goals.

**Objectives:**

The aim of this study was to compare ART outcomes in women with endometriosis following the long GnRH-agonist controlled ovarian stimulation (COS) protocol with those taking the GnRH-antagonist COS protocol.

**Data Sources and Methods:**

MEDLINE, Embase and Web of Science were systematically searched in June 2022. Randomized controlled trials (RCTs) and observational studies comparing the long GnRH-agonist COS protocol and the GnRH-antagonist COS protocol in women with all stages/subtypes of endometriosis were included. Data were synthesized into comprehensive tables for systematic review. The Scottish Intercollegiate Guidelines Network (SIGN) checklists were used for the risk of bias assessment of non-randomized studies and randomized studies, and all the included studies were deemed to have acceptable quality.

**Main Results:**

Eight studies (one RCT and seven observational) with 2695 patients (2761 cycles) were included. Most studies generally reported non-significant differences in clinical pregnancy or live birth rates regardless of the COS protocol used. However, the GnRH-agonist protocol may yield a higher total number of oocytes retrieved, especially mature oocytes. Conversely, the GnRH-antagonist protocol required a shorter COS duration and lower gonadotrophin dose. Adverse outcomes, such as rates of cycle cancellation and miscarriage, were similar between both COS protocols.

**Conclusion:**

Both the long GnRH-agonist and GnRH-antagonist COS protocols generally yield similar pregnancy outcomes. However, the long GnRH-agonist protocol may be associated with a higher cumulative pregnancy rate due to the higher number of retrieved oocytes available for cryopreservation. The underlying mechanisms of the two COS protocols on the female reproductive tract remain unclear. Clinicians should consider treatment costs, stage/subtype of endometriosis and pregnancy goals of their patients when selecting a GnRH analogue for COS. A well-powered RCT is needed to minimize the risk of bias and compare the risk for ovarian hyperstimulation syndrome.

**Registration:**

This review was prospectively registered at PROSPERO under Registration No. CRD42022327604.

## Introduction

Endometriosis is an inflammatory oestrogen-dependent disease characterized by endometrial-like tissue found outside of the uterus. Endometriosis lesions are often located in the peritoneum, ovaries (endometrioma) and uterus, but lesions can also be found in the bowel, urinary tract and vagina. Endometriosis is associated with a wide range of symptoms including visceral syndrome (e.g. pelvic pain, painful urination, dyschezia), dysmenorrhoea and subfertility. Traditionally, endometriosis classification is based on the location of endometrial tissue lesions, and the three most prevalent types are ovarian endometriomas, superficial peritoneal endometriosis or deep endometriosis.^1,2^ Endometriosis is commonly graded on the revised American Society for Reproductive Medicine (r-ASRM) classification scale. Depending on the extent of lesions, it is classified according to the four stages: minimal (stage I), mild (stage II), moderate (stage III) and severe (stage IV).^1,2^

Endometriosis lesions can alter the pelvic anatomy, lead to excess inflammation and can negatively impact the reproductive cycle resulting in subfertility in 30–50% of affected women.^3,4^ In women with endometriosis desiring to become pregnant, around 10–25% require assisted reproductive technology (ART), such as *in vitro* fertilization (IVF) and intracytoplasmic sperm injection (ICSI).^5^ Since the 1980s, the long gonadotrophin-releasing hormone (GnRH) agonist protocol has been the gold standard for controlled ovarian stimulation (COS) to prevent a premature luteinizing hormone (LH) surge and improve ART outcomes. However, this protocol requires an extensive treatment period which is associated with more frequent side effects (such as hot flushes/flashes, bleeding, cyst development and headache) and has a higher risk of ovarian hyperstimulation syndrome (OHSS), which can be life-threatening.^6^ The GnRH-antagonist protocol is a promising alternative with a reduced risk of OHSS, shorter treatment time and often requires a reduced gonadotrophin dose as a result of GnRH antagonists being able to rapidly inhibit GnRH receptors within hours of administration.^7^ However, previous studies report poorer pregnancy outcomes in infertile couples after the GnRH-antagonist protocol.^8,9^

Compared to other causes of infertility, little research has focused on patients with endometriosis specifically, and it remains uncertain whether patients with endometriosis respond similarly to the long GnRH-agonist and GnRH-antagonist COS protocols. Furthermore, the fertilization rate is often overlooked, and it has recently been shown that fertilization rate positively correlates with cumulative live birth rate (LBR).^10^ In this systematic review, we aim to compare ART outcomes following the long GnRH-agonist COS protocol with the GnRH-antagonist COS protocol specifically for women with endometriosis.

## Methods

### Patient populations

The patient populations consisted of women diagnosed with any form of endometriosis undergoing IVF/ICSI with ovarian stimulation using the long GnRH-agonist protocol compared to the GnRH-antagonist protocol.

### Core outcome sets

The primary outcomes were related to pregnancy [clinical pregnancy rate (CPR) and LBR]. Secondary outcomes included the number of oocytes retrieved [total and metaphase II (MII)], fertilization rate, COS parameters (treatment duration and gonadotrophin dose) and adverse ART outcomes (miscarriage rate, cycle cancellation rate and OHSS).

#### Search strategy, eligibility criteria and study selection

**Figure 1.**
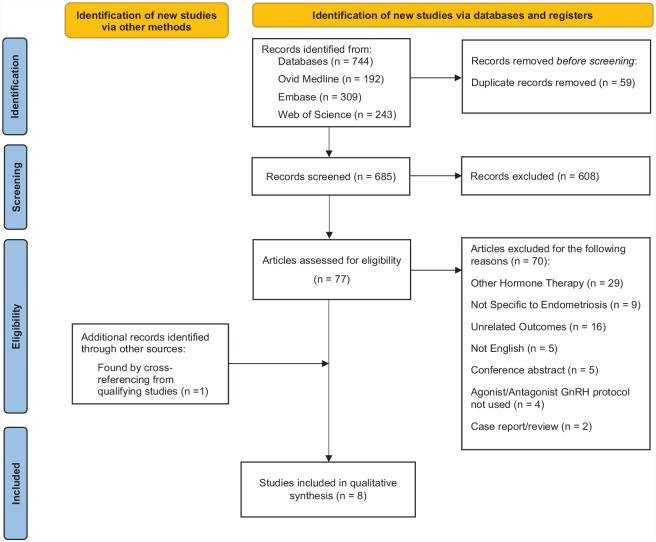
PRISMA flow diagram summarizing the search strategy used to identify qualifying studies.

A systematic search of the published literature up to 10 June 2022 was undertaken on MEDLINE, Embase and Web of Science databases using the Preferred Reporting Items for Systematic reviews and Meta-Analyses (PRISMA) guidelines.^11^ The following keywords and medical subject headings (MeSH) were queried: endometriosis, endometrioma, infertility, GnRH agonist, GnRH antagonist, *in vitro* fertilization and ICSI (the full search strategy is detailed in Supplementary Table 1). Database search results were imported into EndNote (X9, Clarivate Analytics) prior to title and abstract screening. The PRISMA flowchart can be found in Figure 1.

Duplicate studies were removed and two authors (K.K.W.K. and S.O.) independently screened titles and abstracts, and excluded obviously irrelevant studies. Equivocal studies were independently screened by the third author (J.A.T.) until a consensus could be reached. Full manuscripts of studies meeting the selection criteria were retrieved and reviewed by K.K.W.K., S.O. and J.A.T. for the final decision. Studies that used other GnRH-agonist protocols (i.e. ultralong or short) or had patients without endometriosis were excluded. Case reports, conference abstracts with unavailable data and trial protocols were also excluded.

#### Data synthesis and bias assessment

Data extraction was completed by K.K.W.K. and S.O. All data from randomized controlled trials (RCTs) and observational studies (and their relevant subgroups) comparing the long GnRH-agonist protocol *versus* the GnRH-antagonist COS protocol for women with endometriosis were included (Supplementary Table 2). Data were synthesized into outcome tables.

The rigour of study methodology and risk of bias was critically appraised using the relevant Scottish Intercollegiate Guidelines Network (SIGN) criteria for cohort studies and RCTs (description of criteria are available on the SIGN website).^12^ For cohort studies, this tool aims to assess the internal validity (selection of subjects, assessment of exposure and outcomes, confounding factors, statistical analysis) and overall study quality. Statements 1.3, 1.5, 1.6, 1.11 and 1.12 of the SIGN cohort study tool were excluded as all the studies were retrospective in nature and outcomes were objective in accordance with the SIGN’s checklist notes.^12^ The RCT tool assesses for a focused research question, patient randomization, blinding methods, interventions, attrition bias, analysis methods and overall study quality.

## Results

### Study characteristics

Using a systematic searching approach, 744 titles were identified from database searches. After 59 duplicate titles were removed, 608 titles and abstracts were excluded. Ultimately, eight studies were included for the final analysis with a total of 2695 women and 2761 cycles (study characteristics are summarized in Table 1).^13–20^ In these studies, 1721 cycles used the long GnRH-agonist protocol and 1040 cycles used the GnRH-antagonist protocol. Six retrospective analyses,^13–17,20^ one cross-sectional study^18^ and one RCT^19^ were identified. Each study was undertaken at a single centre, and all inclusion/exclusion criteria were available.

**Table 1.** Summary table of characteristics of included endometriosis studies.

Study, Funding	Country	Study design and data collection period	No. of patients/cycles	Endometriosis subtype	Primary outcome	IVF/ICSI	Embryo transfer method	Long GnRH-agonist protocol	GnRH-antagonist protocol	Ovulation trigger method
Zhao *et al.*^13^ 1. Natural Science Foundation of China (81871133); 2. Beijing Municipal Administration of Hospitals Clinical Medicine Development (ZYLX201830)	China	Retrospective cohort study 1 January 2013–30 April 2018	229 total patients and cycles 108 GnRH-agonist cycles 121 GnRH-antagonist cycles	Laparotomy/laparoscopy for unilateral/bilateral ovarian endometrioma and DOR	CPR	IVF	Fresh ET	SC Decapeptyl (0.1 mg) started during luteal phase of prev. cycle	Flexible protocol. SC cetrorelix (0.25 mg/day) started when leading follicle 13–14 mm diameter or after 6 days of Gn.	hCG
Drakopoulos *et al.*^14^ No funding disclosed	Belgium	Retrospective cohort study 2009–2015	386 total patients and cycles 185 GnRH-agonist cycles 201 GnRH-antagonist cycles	Stage I–IV (r-AFS) endometriosis diagnosed laparoscopically	LBR	IVF and ICSI (first cycle only)	Fresh ET	SC/IN triptorelin daily starting d1 or d21 of prev. cycles	Fixed protocol. SC orgalutran (ganirelix) start d6 of cycle.	5000 or 10,000 IU of hCG
Hosseini *et al.*^18^ No funding disclosed	Iran	Cross-sectional study March 2012–November 2015	249 total patients and cycles 129 GnRH-agonist cycles 120 GnRH-antagonist cycles	Stage III–IV (r-AFS)	CPR	ICSI (first cycle only)	Fresh ET	SC triptorelin (0.1 mg/day) start d21 luteal phase of prev. cycle	Fixed protocol. SC cetrorelix (0.25 mg/dl) start d6 of cycle.	5000 or 10,000 IU of hCG
Kolanska *et al.*^20^ (France) No funding disclosed	France	Retrospective cohort study January 2013–May 2014 (GnRH antagonist) and June 2014–May 2015 (GnRH agonist)	218 total patients and 284 total cycles 165 GnRH-agonist cycles 119 GnRH-antagonist cycles	Endometriosis diagnosed based on Hx, physical examination, transvaginal ultrasound examination confirmed by MRI and surgery	CPR	IVF and ICSI	Fresh and frozen ET	GnRH agonist not specified, dose not given	GnRH antagonist not specified, dose not given.	hCG or triptorelin
Bastu *et al.*^15^ Scientific Research Projects Coordination Unit of Istanbul University (#33502)	Turkey	Retrospective cohort study 1 January 2002–1 January 2012	86 total patients and cycles 44 GnRH-agonist cycles 42 GnRH-antagonist cycles	Stage III–IV (r-AFS) endometriosis with large unilateral/bilateral endometriomas > 4 cm	Unspecified	IVF and ICSI (first cycle only)	Fresh ET	SC triptorelin (0.1 mg/day) or leuprolide (0.5 mg/day) start d21 luteal phase of prev. cycle	Flexible protocol. SC cetrorelix (0.25 mg/day) started when leading follicle 12–13 mm diameter.	hCG
Rodriguez-Purata *et al.*^16^ No funding disclosed	Spain	Retrospective cohort study January 2000–December 2010	1180 total patients and cycles 919 GnRH-agonist cycles 261 GnRH-antagonist cycles	Stage I–IV (AFS) endometriosis confirmed surgically or by ultrasound	CPR	IVF and ICSI	Fresh ET	Leuprolide acetate start d20 of prev. cycle	Flexible protocol. Ganirelix or cetrorelix (0.25 mg/day) started when 14 mm follicle visualized. OCP started d1–3 of prev. cycle taken for 14–24 days followed by 3- to 5-day washout period before starting COS.	hCG
Ruggiero *et al.*^17^ No funding disclosed	Italy	Retrospective cohort January 2007–June 2009	101 total patients and cycles 49 GnRH-agonist cycles 52 GnRH-antagonist cycles	Stage III–IV (ASRM) endometriosis with (Hx operative laparoscopy)	CPR	IVF and ICSI	Fresh ET	SC leuprorelin (1 mg–0.2 ml/day) start d21 luteal phase of prev. cycle	Flexible protocol. SC cetrorelix (0.25 mg/day) started when leading follicle 14 mm diameter.	hCG
Pabuccu *et al.*^19^ No funding disclosed	Turkey	Prospective RCT November 2002–February 2006	246 total patients and cycles 122 GnRH-agonist cycles 124 GnRH-antagonist cycles	Stage I–IV (r-AFS) endometriosis confirmed laparoscopically: Group 1 = Stage I–II Group 2 = Hx ovarian surgery for endometrioma without recurrence Group 3 = uni/bilateral endometrioma(s)	CPR	ICSI (first cycle only)	Fresh ET	SC triptorelin (0.1 mg/day) start d21 luteal phase of prev. cycle	Flexible protocol. SC cetrorelix (0.25 mg/day) daily until hCG injection. Started when leading follicle 14 mm diameter and serum E_2_ > 600 pg/ml.	hCG

♀, female; #, number; ASRM, American Society for Reproductive Medicine Classification; COS, controlled ovarian stimulation; CP, clinical pregnancy; CPR, clinical pregnancy rate; d, days; DOR, diminished ovarian reserve; ET, embryo transfer; FR, fertilization rate; GnRH-a, gonadotrophin-releasing hormone agonist; GnRH-ant, gonadotrophin-releasing hormone antagonist; Hx, history; hMG, human menopausal gonadotrophin; IN, intranasal; ICSI, intracytoplasmic sperm injection; IR, implantation rate; IU, units; IVF, *in vitro* fertilization; MRI, magnetic resonance imaging; N, number of patients; OCP, oral contraceptive pills; OR, oocytes retrieved; PCOS, polycystic ovarian syndrome; Prev., previous; r-AFS, revised American Fertility Society classification system; RCT, randomised controlled trial; SC, subcutaneous.

For the long GnRH-agonist protocol, four studies administered triptorelin,^14,15,18,19^ two studies administered leuprorelin^16,17^ and one study administered decapeptyl^13^ daily starting from day 20 to 21 of the previous menstrual cycle. One study^20^ did not specify which GnRH agonist was used and started treatment after day 21 of the preceding cycle. Seven studies in the GnRH-antagonist arm administered subcutaneous cetrorelix or ganirelix.^13–19^ Five of which followed a flexible multiple dosing protocol^13,15–17,19^ and two of which followed a fixed protocol from day 5 or day 6.^14,18^ One study also gave patients in the GnRH-antagonist arm an oral contraceptive pill pretreatment taken for 14–24 days in the preceding cycle followed by a 3- to 5-day washout period.^16^ One study did not specify the antagonist used and started the protocol after at least 6 weeks of oral contraceptives.^20^

### Study quality and risk of bias assessment

The completed SIGN assessments for observational studies and the RCT can be found in Tables 2 and 3, respectively. Since the study by Hosseini *et al.* was a cross-sectional study, a SIGN^12^ checklist was not required (as described by SIGN’s study design algorithm). As mentioned earlier, Statements 1.3, 1.5, 1.6, 1.11 and 1.12 were not applicable for retrospective studies. Statements 1.3, 1.8 and 1.9 did not apply since patients did not have the outcome before starting the intervention (1.4) and the primary outcomes of interest (pregnancy and LBRs) were objective and would not be affected by blinding (1.8 and 1.9). All studies had a clearly focused question, had representative patient characteristics and clearly defined outcomes. All studies had overall acceptable quality and were eligible for review. Two observational studies mentioned that the assignment of the GnRH-agonist protocol or the GnRH-antagonist protocol varied between clinicians.^15,16^ Kolanska *et al.*^20^ were the only observational study to exclusively offer either the GnRH-agonist protocol or the antagonist protocol during specific timelines minimizing selection bias to either protocol. Although Rodriguez-Purata *et al.*^16^ mentioned that poorer responders tended to use the antagonist protocol, a propensity score matching statistical method was used to compare CPRs. This method adjusts for covariates such as disease severity and comorbidities that may affect the probability of patients allocated to a certain treatment. As such, only patients with similar characteristics were compared for this outcome which helped mitigate selection bias. Two studies performed multivariate logistic regression to identify predictive factors affecting pregnancy or birth rates.^13,18^ The inclusion of a small number of women with polycystic ovary syndrome, tubal infertility or adenomyosis alongside endometriosis also raised concerns for additional confounding factors.^14,20^ Four of the studies only included women undergoing their first IVF/ICSI cycle, which reduced the risk of confounders from women who require multiple IVF cycles due to poorer ART outcomes.^14,15,18,19^ Since the primary outcomes of interest were objective, the studies were at lower risk of measurement bias. For the RCT, randomization methods were adequate, although there was a lack of blinding. An adequate sample size for pretest power estimation could not be calculated since there was a lack of studies comparing the long GnRH-agonist *versus* the GnRH-antagonist protocol prior to this RCT.^19^

**Table 2.** Quality of evidence for GnRH-agonist and GnRH-antagonist COS protocols in endometriosis observational studies using the SIGN checklist.

Author year	Question	Bastu *et al*.^15^	Drakopoulos *et al.*^14^	Kolanska *et al.*^20^	Rodriguez-Purata *et al*.^16^	Ruggiero *et al*.^17^	Zhao *et al.*^13^
Section 1: Internal validity							
1.1	The study addresses an appropriate and clearly focused question	Yes	Yes	Yes	Yes	Yes	Yes
1.2	The two groups being studied are selected from source populations that are comparable in all respects other than the factor under investigation	Yes	Yes	Yes	Yes	Yes	Yes
1.4	The likelihood that some eligible subjects might have the outcome at the time of enrolment is assessed and taken into account in the analysis	Does not apply	Does not apply	Does not apply	Does not apply	Does not apply	Does not apply
1.7	The outcomes are clearly defined	Yes	Yes	Yes	Yes	Yes	Yes
1.8	The assessment of outcome is made blind to exposure status. If the study is retrospective, this may not be applicable	Does not apply	Does not apply	Does not apply	Does not apply	Does not apply	Does not apply
1.9	Where blinding was not possible, there is some recognition that knowledge of exposure status could have influenced the assessment of outcome	Does not apply	Does not apply	Does not apply	Does not apply	Does not apply	Does not apply
1.10	The method of assessment of exposure is reliable	Yes	Yes	Yes	Yes	Yes	Yes
1.13	The main potential confounders are identified and taken into account in the design and analysis	Cannot say	Cannot say	Yes	Yes	Cannot say	Yes
1.14	Have confidence intervals been provided?	No	No	No	No	No	No
Total fulfilment (out of 6)		4	4	5	5	4	5
Section 2: Overall assessment of the study							
2.1	How well was the study done to minimize the risk of bias or confounding?	Acceptable	Acceptable	Acceptable	Acceptable	Acceptable	Acceptable
2.2	Taking into account clinical considerations, your evaluation of the methodology used and the statistical power of the study, do you think there is clear evidence of an association between exposure and outcome?	Cannot say	No	Cannot say	No	No	No
2.3	Are the results of this study directly applicable to the patient group targeted in this guideline?	Yes	Yes	Yes	Yes	Yes	Yes

**Table 3.** Quality of evidence for GnRH-agonist and GnRH-antagonist COS protocols in endometriosis RCTs using the SIGN checklist.

Author year	Question	Pabuccu *et al*.^19^
Section 1: Internal validity		
1.1	The study addresses an appropriate and clearly focused question	Yes
1.2	The assignment of subjects to treatment groups is randomized	Yes
1.3	An adequate concealment method is used	Cannot say
1.4	The design keeps subjects and investigators ‘blind’ about treatment allocation	No
1.5	The treatment and control groups are similar at the start of the trial	Yes
1.6	The only difference between groups is the treatment under investigation	Yes
1.7	All relevant outcomes are measured in a standard, valid and reliable way	Yes
1.8	What percentage of the individuals or clusters recruited into each treatment arm of the study dropped out before the study was completed?	Cannot say
1.9	All the subjects are analysed in the groups to which they were randomly allocated	No
1.10	Where the study is carried out at more than one site, results are comparable for all sites	Does not apply
Section 2: Overall assessment of the study		
2.1	How well was the study done to minimize bias?	Acceptable
2.2	Taking into account clinical considerations, your evaluation of the methodology used and the statistical power of the study, are you certain that the overall effect is due to the study intervention?	Cannot say
2.3	Are the results of this study directly applicable to the patient group targeted in this guideline?	Yes

### ART outcomes

#### Clinical pregnancy rate

CPR was reported by all eight studies and was calculated by CPR per embryo transfer (ET) in three studies^13,17,18^ or CPR per patient/cycle in four studies,^14–16,19^ (see Table 4). Kolanska *et al.*^20^ were the only study to report both CPR per cycle with ET and CPR per patient and analysed fresh/frozen ETs separately. Most studies found no significant difference in CPR^13–20^ between the long GnRH-agonist and GnRH-antagonist protocols except for two subgroup analyses.^18,20^ For advanced endometriosis, Hosseini *et al.*^18^ reported a significantly higher pregnancy rate with the GnRH agonist when anti-Müllerian hormone (AMH) levels were between 1.1 and 2.7 ng/ml (*p* = 0.04). Kolanska *et al.*^20^ found significantly higher CPR per started cycle with the GnRH agonist when analysing fresh ETs from women with all forms of endometriosis combined (*p* = 0.02) but no significant difference for CPR per cycle with ET only or freeze–thaw cycles. No difference (*p* > 0.05) was found when analysing deep or ovarian endometriosis in isolation regardless of fresh or freeze–thaw cycles.^20^ Multivariate logistic regression analysis was performed by two studies which identified maternal age (*p* = 0.006) and number of embryos (*p* = 0.03) as main factors that may predict pregnancy rate.^13,18^

**Table 4.** CPR outcome data for GnRH-agonist and GnRH-antagonist COS protocols in women with endometriosis.

Study (# of patients)	Method of calculating CPR	GnRH agonist	GnRH antagonist	*p* value
Bastu *et al.*^15^ 86 patients	CPR/patient	20.5 (9/44)	19.1 (8/42)	NS
Zhao *et al.*^13^ 229 patients	CPR/ET cycle	28.99 (20)	33.33 (29)	NS
Drakopoulos *et al.*^14^ 386 patients	CPR/patient	Stage I–II 50 (21/42) Stage III–IV 34.3 (49/143)	Stage I–II 36 (27/75) Stage III–IV 32.5 (41/126)	Stage I–II 0.14 Stage III–IV 0.7
Hosseini *et al.*^18^ 249 patients	CPR/ET	AMH < 1.1 5.5 (2/36) 1.1 ⩽ AMH ⩽ 2.7 41.3 (19/46) AMH > 2.7 17.6 (6/34)	AMH < 1.1 13.6 (6/36) 1.1 ⩽ AMH ⩽ 2.7 20.9 (9/43) AMH > 2.7 39.4 (13/33)	AMH < 1.1 0.2 1.1 ⩽ AMH ⩽ 2.7 0.04 AMH > 2.7 0.06
Kolanska *et al.*^20^ 218 patients	CPR/cycle and CPR/ET	All endometriosis Fresh embryos CPR/cycle: 25 (41/165) CPR/ET: 29 (41/165) Freeze–thaw embryos CPR/cycle: 5 (8/165) CPR/ET: 16 (8/165) Fresh + frozen ET CPR/cycle: 29 (48/165) CPR/ET: 29 (48/165) DE without either endometrioma or adenomyosis Fresh CPR/cycle: 28 (7/25) CPR/ET: 30 (7/25) Freeze–thaw CPR/cycle: 0 (0/25) CPR/ET: 0 (0/10) Fresh + frozen ET CPR/cycle: 28 (7/25) CPR/ET: 29 (7/24) DE with endometrioma but without adenomyosis Fresh CPR/cycle: 28 (14/50) CPR/ET: 31 (14/45) Freeze–thaw CPR/cycle: 4 (2/50) CPR/ET: 11 (2/18) Fresh + frozen ET CPR/cycle: 30 (15/50) CPR/ET: 33 (15/46) Endometrioma alone Fresh CPR/cycle: 7 (1/14) CPR/ET: 10 (1/10)	All endometriosis Fresh embryos CPR/cycle: 13 (15/119) CPR/ET: 17 (15/119) Freeze–thaw embryos CPR/cycle: 7 (8/119) CPR/ET: 22 (8/119) Fresh + frozen ET CPR/cycle: 18 (22/119) CPR/ET: 18 (22/119) DE without either endometrioma or adenomyosis Fresh CPR/cycle: 6 (1/16) CPR/ET: 10 (1/16) Freeze–thaw CPR/cycle: 6 (1/16) CPR/ET: 20 (1/16) Fresh + frozen ET CPR/cycle: 13 (2/16) CPR/ET: 18 (2/11) DE with endometrioma but without adenomyosis Fresh CPR/cycle: 14 (5/36) CPR/ET: 17 (5/29) Freeze–thaw CPR/cycle: 8 (3/36) CPR/ET: 23 (3/13) Fresh + frozen ET CPR/cycle: 22 (8/36) CPR/ET: 24 (8/33) Endometrioma alone Fresh CPR/cycle: 22 (2/9) CPR/ET: 29 (2/7)	All endometriosis Fresh embryos CPR/cycle: 0.017 CPR/ET: 0.053 Freeze–thaw embryos CPR/cycle: 0.70 CPR/ET: 0.70 Fresh + frozen ET CPR/cycle: 0.06 CPR/ET: 0.10 DE without either endometrioma or adenomyosis Fresh CPR/cycle: 0.0865 CPR/ET: 0.2081 Freeze–thaw CPR/cycle: 0.2057 CPR/ET: 0.1432 Fresh + frozen ET CPR/cycle: 0.2421 CPR/ET: 0.4900 DE with endometrioma but without adenomyosis Fresh CPR/cycle: 0.1197 CPR/ET: 0.1824 Freeze–thaw CPR/cycle: 0.3969 CPR/ET: 0.3714 Fresh + frozen ET CPR/cycle: 0.4214 CPR/ET: 0.3714 Endometrioma alone Fresh CPR/cycle: 0.2946 CPR/ET: 0.3229
		Freeze–thaw CPR/cycle: 14 (2/14) CPR/ET: 67 (2/3) Fresh + frozen ET CPR/cycle: 21 (3/14) CPR/ET: 27 (3/11) Endometriosis without adenomyosis Fresh CPR/cycle: 25 (27/109) CPR/ET: 28 (27/95) Freeze–thaw CPR/cycle: 5 (5/109) CPR/ET: 14 (5/35) Fresh + frozen ET CPR/cycle: 28 (31/109) CPR/ET: 32 (31/98)	Freeze–thaw CPR/cycle: 0 (0/9) CPR/ET: 0 (0/0) Fresh + frozen ET CPR/cycle: 22 (2/9) CPR/ET: 29 (2/7) Endometriosis without adenomyosis Fresh CPR/cycle: 12 (10/86) CPR/ET: 15 (10/65) Freeze–thaw CPR/cycle: 7 (6/86) CPR/ET: 23 (6/26) Fresh + frozen ET CPR/cycle: 17 (15/86) CPR/ET: 21 (15/70)	Freeze–thaw CPR/cycle: 0.2354 CPR/ET: NA Fresh + frozen ET CPR/cycle: 0.9641 CPR/ET: 0.9522 Endometriosis without adenomyosis Fresh CPR/started: 0.0201 CPR/ET: 0.0548 Freeze–thaw CPR/cycle: 0.4727 CPR/ET: 0.3771 Fresh + frozen ET CPR/cycle: 0.0725 CPR/ET: 0.1437
Rodriguez-Purata *et al.*^16^ 1180 patients	CPR/cycle	Group 1 = 41.9 Group 2 = 39.7 Group 3 = 15.4	Group 1 = 30 Group 2 = 36.4 Group 3 = 18.9	Group 1 = 0.475 Group 2 = 0.77 Group 3 = 0.716
Ruggiero *et al.*^17^ 101 patients	CPR/ET	16.7	19.3	NS
Pabuccu *et al.*^19^ 246 patients	CPR/patient	Stage I–II 31.2 (15/48) Hx endometrioma without recurrence 39 (16/41) Uni/bilateral endometrioma 24.2 (8/33)	Stage I–II 30 (15/50) Hx endometrioma without recurrence 27.5 (11/40) Uni/bilateral endometrioma 20.5 (7/34)	Stage I–II NS Hx endometrioma without recurrence NS Uni/bilateral endometrioma NS

AMH, anti-Müllerian hormone; CPR, clinical pregnancy rate; DE, deep endometriosis; ET, embryo transfer; Hx, history; NS, not statistically significant.

All values shown as percentage (absolute number).

#### Live birth rate

LBR was included in three studies^13,14,20^ (see Table 5). The LBR was calculated as either births per ET cycles^13,20^ or births per started cycle regardless of the number of embryos transferred.^14,20^ Two studies found no significant difference in LBR between protocols.^13,14^ Kolanska *et al.* performed subgroup analyses by endometriosis subtype and fresh/freeze–thaw embryos and found no significant difference between protocols in LBR for patients with DE or endometriomas in isolation regardless of ET methods. However, the LBR per started cycle (regardless of whether embryos were transferred) was significantly higher (*p* = 0.02) in the long GnRH-agonist group.^20^ Zhao *et al.*^13^ were the only study to perform regression analysis and found maternal age to be the strongest predictive factor for women with diminished ovarian reserve (DOR) following ovarian cystectomy.

**Table 5.** LBR outcome data for GnRH-agonist and GnRH-antagonist COS protocols in women with endometriosis.

Study	Method of calculating LBR	GnRH agonist	GnRH antagonist	*p* value
Zhao *et al.*^13^ 229 patients	Birth/ET cycle	24.64 (17)	19.54 (17)	NS
Drakopoulos *et al.*^14^ 386 patients	Birth/patient	Stage I–II 42.8 (18) Stage III–IV 27.3 (39)	Stage I–II 26.7 (20) Stage III–IV 23.8 (30)	Stage I–II 0.07 Stage III–IV 0.5
Kolanska *et al.*^20^ 218 patients	Birth/ET	All endometriosis Fresh embryos LBR/cycle: 18 (31/165) LBR/ET: 22 (31/165) Freeze–thaw embryos LBR/cycle: 2 (3/165) LBR/ET: 6 (3/165) Fresh + frozen ET LBR/cycle: 21 (34/165) LBR/ET: 24 (34/165) DE without either endometrioma or adenomyosis Fresh LBR/cycle: 20 (5/25) LBR/ET: 22 (5/23) Freeze–thaw LBR/cycle: 0 (0/25) LBR/ET: 0 (0/10) Fresh + frozen ET LBR/cycle: 20 (5/25) LBR/ET: 21 (5/24) DE with endometrioma but without adenomyosis Fresh LBR/cycle: 18 (9/50) LBR/ET: 20 (9/45) Freeze–thaw LBR/cycle: 2 (1/50) LBR/ET: 6 (1/18) Fresh + frozen ET LBR/cycle: 20 (10/50) LBR/ET: 22 (10/46) Endometrioma alone Fresh LBR/cycle: 0 (0/14) LBR/ET: 0 (0/10) Freeze–thaw LBR/cycle: 7 (1/14) LBR/ET: 33 (1/3) Fresh + frozen ET LBR/cycle: 7 (1/14) LBR/ET: 9 (1/11) Endometriosis without adenomyosis Fresh LBR/cycle: 15 (16/109) LBR/ET: 17 (16/95) Freeze–thaw LBR/cycle: 2 (2/109) LBR/ET: 6 (2/35) Fresh + frozen ET LBR/cycle: 17 (18/109) LBR/ET: 18 (18/98)	All endometriosis Fresh embryos LBR/cycle: 8 (9/119) LBR/ET: 10 (9/119) Freeze–thaw embryos LBR/cycle: 7 (8/119) LBR/ET: 22 (8/119) Fresh + frozen ET LBR/cycle: 14 (17/119) LBR/ET: 18 (17/119) DE without either endometrioma or adenomyosis Fresh LBR/cycle: 0 (0/16) LBR/ET: 0 (0/10) Freeze–thaw LBR/cycle: 6 (1/16) LBR/ET: 20 (1/5) Fresh + frozen ET LBR/cycle: 6 (1/16) LBR/ET: 9 (1/11) DE with endometrioma but without adenomyosis Fresh LBR/cycle: 6 (2/36) LBR/ET: 7 (2/29) Freeze–thaw LBR/cycle: 0 (0/36) LBR/ET: 0 (0/13) Fresh + frozen ET LBR/cycle: 6 (2/36) LBR/ET: 6 (2/33) Endometrioma alone Fresh LBR/cycle: 11 (1/9) LBR/ET: 14 (1/7) Freeze–thaw LBR/cycle: 0 (0/9) LBR/ET: 0 (0/0) Fresh + frozen ET LBR/cycle: 11 (1/9) LBR/ET: 14 (1/7) Endometriosis without adenomyosis Fresh LBR/cycle: 5 (4/86) LBR/ET: 6 (4/65) Freeze–thaw LBR/cycle: 1 (1/86) LBR/ET: 4 (1/26) Fresh + frozen ET LBR/cycle: 7 (6/86) LBR/ET: 9 (6/70)	All endometriosis Fresh embryos LBR/cycle: 0.04 LBR/ET: 0.02 Freeze–thaw embryos LBR/cycle: 0.09 LBR/ET: 0.001 Fresh + frozen ET LBR/cycle: 0.19 LBR/ET: 0.29 DE without either endometrioma or adenomyosis Fresh LBR/cycle: 0.0563 LBR/ET: 0.1095 Freeze–thaw LBR/cycle: 0.2057 LBR/ET: 0.1432 Fresh + frozen ET LBR/cycle: 0.2243 LBR/ET: 0.3922 DE with endometrioma but without adenomyosis Fresh LBR/cycle: 0.0883 LBR/ET: 0.1219 Freeze–thaw LBR/cycle: 0.3934 LBR/ET: 0.3877 Fresh + frozen ET LBR/cycle: 0.0565 LBR/ET: 0.0555 Endometrioma alone Fresh LBR/cycle:0.2022 LBR/ET:0.2179 Freeze–thaw LBR/cycle: 0.4123 LBR/ET: NA Fresh + frozen ET LBR/cycle: 0.7417 LBR/ET: 0.7324 Endometriosis without adenomyosis Fresh LBR/cycle: 0.0219 LBR/ET: 0.0447 Freeze–thaw LBR/cycle:0.7050 LBR/ET: 0.7386 Fresh + frozen ET LBR/cycle: 0.0441 LBR/ET: 0.0736

All values shown as percentage (absolute number).

CPR, clinical pregnancy rate; DE, deep endometriosis; ET, embryo transfer; LBR, live birth rate; NS, not statistically significant.

#### Number of oocytes retrieved

Six studies assessed the total number of oocytes retrieved^13,14,16–19^ (see Table 6). Two studies included women with resected endometrioma and found no difference in the number of oocytes retrieved between COS protocols.^13,19^ However, in women with active endometriomas, Pabuccu *et al.* reported a higher number of oocytes (*p* = 0.002) retrieved using the GnRH-agonist protocol. The number of oocytes retrieved from patients with stage I–II endometriosis was reported by two studies, and both found no significant difference between protocols.^14,19^ Three studies included women with stage III–IV endometriosis and two found no significant difference.^14,17^ In a subgroup analysis of advanced endometriosis grouped by AMH levels, women with AMH levels between 1.1 and 2.7 ng/ml did not differ in the number of oocytes retrieved between the two COS protocols. However, in women with AMH less than 1.1 ng/ml, the long GnRH-agonist protocol yielded more oocytes, while in women with AMH greater than 2.7 ng/ml, the GnRH-antagonist protocol led to an increased number of oocytes retrieved.^18^ Rodriguez-Purata *et al.*^16^ included all stages of endometriosis and found a significantly higher number of oocytes retrieved using the long GnRH-agonist protocol (*p* = 0.001). However, the propensity score was not applied to this outcome.

**Table 6.** Number of oocytes retrieved for GnRH-agonist and GnRH-antagonist COS protocols in women with endometriosis.

Study (# of patients)	GnRH agonist	GnRH antagonist	*p* value
Total number of oocytes retrieved			
Zhao *et al.*^13^ 229 patients	4.13 ± 2.04	3.67 ± 1.92	NS
Drakopoulos et al.*^14^ 386 patients	Stage I–II 9 (6–13) Stage III–IV 8 (5–11)	Stage I–II 7 (5–12) Stage III–IV 7 (5–11)	Stage I–II 0.09 Stage III–IV 0.33
Hosseini *et al.*^18^ 249 patients	AMH < 1.1 3.04 ± 1.22 1.1 ⩽ AMH ⩽ 2.7 8.07 ± 3.36 AMH > 2.7 11.3 ± 3.02	AMH < 1.1 2.3 ± 1.72 1.1 ⩽ AMH ⩽ 2.7 6.8 ± 3.36 AMH > 2.7 13.5 ± 3.6	AMH < 1.1 0.03 1.1 ⩽ AMH ⩽ 2.7 0.08 AMH > 2.7 0.01
Rodriguez-Purata *et al.*^16^ 1180 patients	11.2 ± 6.6	6.7 ± 4.4	0.001
Ruggiero *et al.*^17^ 101 patients	3.8 ± 2.7	4.8 ± 3	0.15
Pabuccu *et al.*^19^ 246 patients	Stage I–II 13.3 ± 5.9 Hx endometrioma without recurrence 10.4 ± 5.9 Uni/bilateral endometrioma 8.2 ± 5.5	Stage I–II 8.9 ± 4.4 Hx endometrioma without recurrence 8.3 ± 4.5 Uni/bilateral endometrioma 6.7 ± 2.6	Stage I–II NS Hx endometrioma without recurrence NS Uni/bilateral endometrioma 0.002
Number of mature (MII) oocytes retrieved			
Bastu *et al.*^15^ 86 patients	7.93 ± 5.43	5.25 ± 5.51	0.001
Rodriguez-Purata *et al.*^16^ 1180 patients	8.3 ± 5.3	5.3 ± 3.6	0.001
Ruggiero *et al.*^17^ 101 patients	3.3 ± 0.78	5.3 ± 3.6	0.001
Pabuccu *et al.*^19^ 246 patients	Stage I–II 9.6 ± 4.5 Hx endometrioma without recurrence 8.8 ± 4.6 Uni/bilateral endometrioma 6.5 ± 4.2	Stage I–II 8.9 ± 4.4 Hx endometrioma without recurrence 4.3 ± 2.6 Uni/bilateral endometrioma 4.9 ± 1.6	Stage I–II NS Hx endometrioma without recurrence 0.0001 Uni/bilateral endometrioma 0.01

#, number; AMH, anti-Müllerian hormone; Hx, history; IQR, interquartile range; NS, not statistically significant.

All number of oocytes retrieved shown as mean ± standard deviation unless * [mean (IQR)].

Four studies included the number of MII oocytes retrieved^15–17,19^ (see Table 6). Pabuccu *et al.* were the only study to analyse patients with stage I–II endometriosis and found no difference between the two protocols. In severe stages of endometriosis, Ruggiero *et al.*^17^ reported a significantly higher number of MII oocytes retrieved when the GnRH-antagonist protocol was used. Two studies included patients with active/resected endometrioma and found a significantly higher number of MII oocytes retrieved when GnRH-agonist COS was used (*p* = 0.0001–0.01).^15,19^ Rodriguez-Purata *et al.*^16^ did not apply the propensity score matching for this outcome but also found a significantly higher number of MII oocyte yield using the GnRH-agonist protocol.

#### Fertilization rate

Fertilization rate (FR) was reported by four studies^13,15,17,19^ (see Table 7). Pabuccu *et al.*^19^ were the only study to compare FR in women with stage I–II endometriosis and found no difference between COS protocols. Also, no significant difference was found in women with severe endometriosis.^17^ Two observational studies of women with endometrioma resection^13,15^ reported no significant difference in FR although the RCT found a significantly higher FR when the long GnRH-agonist was used in resected endometrioma (*p* = 0.001) but not in active endometrioma.^19^

**Table 7.** Fertilization rate outcome data for GnRH-agonist and GnRH-antagonist COS protocols in women with endometriosis.

Study	GnRH agonist	GnRH antagonist	*p* value
Bastu *et al.*^15^ 86 patients	75.75 ± 32.98	71.32 ± 32.94	NS
Zhao *et al.*^13^ 229 patients	78.46 ± 24.78	73.52 ± 28.92	NS
Ruggiero *et al.*^17^ 386 patients	76.9	83.4	NS
Pabuccu *et al.*^19^ 246 patients	Stage I–II 76.4 ± 18.9 Hx endometrioma without recurrence 71.2 ± 22.4 Uni/bilateral endometrioma 75.6 ± 15.4	Stage I–II 73.7 ± 22.7 Hx endometrioma without recurrence 63.9 ± 21.1 Uni/bilateral endometrioma 73.5 ± 23.7	Stage I–II NS Hx endometrioma without recurrence 0.001 Uni/bilateral endometrioma NS

Hx, history; NS, not statistically significant.

All values shown as mean ± standard deviation.

### COS parameters

#### COS duration

Among the seven studies that reported COS duration^13–17,19,20^ (see Table 8), only one found a significant difference in the COS duration^14^ with the agonist protocol having a longer duration compared to the antagonist protocol (*p* = 0.001). Drakopoulos *et al.*^14^ found a significant difference between the GnRH-agonist and GnRH-antagonist duration in women with stage III–IV endometriosis (*p* < 0.001) but no difference in women with stage I–II endometriosis.

**Table 8.** Summary of COS parameters for GnRH-agonist and GnRH-antagonist COS protocols in women with endometriosis.

Study (# of patients)	Method of calculating COS dose and duration	GnRH agonist	GnRH antagonist	*p* value
COS duration				
Bastu *et al.*^15^ 86 patients	Mean (days) ± SD	11.00 ± 2.13	10.16 ± 1.98	NS
Zhao *et al.*^13^ 229 patients	Mean (days) ± SD	10.08 ± 2.22	9.83 ± 1.74	NS
Drakopoulos *et al.*^14^ 386 patients	Mean (days) (IQR)	Stage I–II 11 (9–12) Stage III–IV 11 (9–12)	Stage I–II 9 (8–11) Stage III–IV 9 (8–11)	Stage I–II 0.1 Stage III–IV <0.001
Kolanska *et al.*^20^ 218 patients	Mean (days) ± SD	11 (6–92)	11 (6–18)	0.3
Rodriguez-Purata *et al.*^16^ 1180 patients	Mean (days) ± SD	10.5 ± 2.1	10.16 ± 1.98	NS
Ruggiero *et al.*^17^ 101 patients	Mean (days) ± SD	11.8 ± 1.6	11.0 ± 1.7	0.09
Pabuccu *et al.*^19^ 246 patients	Mean (days) ± SD	Stage I–II 10.1 ± 1.4 Hx endometrioma without recurrence 11.2 ± 1.5 Uni/bilateral endometrioma 10.5 ± 1.6	Stage I–II 9.9 ± 1.2 Hx endometrioma without recurrence 10.5 ± 1.2 Uni/bilateral endometrioma 9.9 ± 1.4	NS
COS dose				
Bastu *et al.*^15^ 86 patients	Mean [dose (IU)] ± SD	3167.0 ± 1124.4	3261.1 ± 1653.9	NS
Zhao *et al.*^13^ 229 patients	Mean [dose (IU)] ± SD	2594.24 ± 1057.56	2581.61 ± 827.11	NS
Drakopoulos *et al.*^14^ 386 patients	Median [dose (IU)] (IQR)	Stage I–II 2025 (1800–2575) Stage III–IV 2400 (2000–3000)	Stage I–II 1650 (1200–2400) Stage III–IV 2000 (1350–2625)	Stage I–II <0.001 Stage III–IV <0.001
Kolanska *et al.*^20^ 218 patients	Median (dose [IU]) (range)	2425 (30–6600)	2500 (14–5850)	0.4
Rodriguez-Purata *et al.*^16^ 1180 patients	Mean (dose [IU]) ± SD	2800 ± 1106	3261.1 ± 1653.9	NS
Ruggiero *et al.*^17^ 101 patients	Mean [dose (IU)] ± SD	4817 ± 894	3923 ± 777	0.05
Pabuccu *et al.*^19^ 246 patients	Mean (ampoules) ± SD	Stage I–II 28.6 ± 8.7 Hx endometrioma without recurrence 32.1 ± 9.3 Uni/bilateral endometrioma 30.3 ± 8.7	Stage I–II 27.4 ± 8.8 Hx endometrioma without recurrence 29.9 ± 8.5 Uni/bilateral endometrioma 28.2 ± 8.7	NS

COS, controlled ovarian stimulation; Hx, history; IQR, interquartile range; SD, standard deviation.

#### Gonadotrophin dose

In the majority of the papers reviewed, there were no significant differences in the total gonadotrophin dose (IU) required for COS treatment between the two protocols (see Table 8). Drakopoulos *et al.*^14^ reported that both women with stage I–II and stage III–IV endometriosis required a greater gonadotrophin dose when using the long GnRH-agonist protocol (*p* < 0.001) as opposed to the GnRH-antagonist protocol. Ruggerio *et al.*^17^ also found that the gonadotrophin dose between the two protocols was greater in the agonist arm (*p* = 0.05) when observing women with stage III–IV endometriosis. Whereas two studies that only included women with resected endometriomas found no difference in gonadotrophin dose between the protocols.^13,15^

Pabuccu *et al.*^19^ reported the amount of gonadotrophin used by the number of recombinant follicle-stimulating hormone (FSH) ampoules and no significant differences were observed in women with stage I–II endometriosis, resected endometriomas or active endometriomas.

### Adverse ART cycle outcomes

The risk of developing OHSS was not explicitly reported as an outcome in any of the studies. The miscarriage rate was reported by three studies but there was no significant difference between the outcomes of the GnRH-agonist or antagonist protocols^17,19,20^ (see Table 9). Pabuccu *et al.* observed no significant difference between the two protocols in the miscarriage rate in women with stage I–II endometriosis, resected endometrioma or active endometrioma. Interestingly, this study included the total number of cycle cancellations due to the risk of developing OHSS or insufficient ovarian response but did not specify how the cancellations were distributed between the two protocols nor were *p*-values specified.^19^ Three papers measured the cycle cancellation rate in patients taking these two protocols and found that cycles were cancelled due to a variety of reasons, including insufficient ovarian response, risk of ovarian hyperstimulation, elevated progesterone levels and a low number of oocytes or embryos.^17,18,20^ Of these three studies, all found that the cancellation rate did not differ significantly between the two protocols.

**Table 9.** Summary of adverse ART outcome data for GnRH-agonist and GnRH-antagonist COS protocols in women with endometriosis.

Study (# of patients)	Data presentation	GnRH agonist	GnRH antagonist	*p* value
Cycle cancellation rate				
Hosseini *et al*.^18^ 249 patients	Percentage (absolute number)	AMH < 1.1 CCR: 26.53 (13/49) 1.1 ⩽ AMH ⩽ 2.7 CCR: 0 (0/0) AMH > 2.7 CCR: 0 (0/0)	AMH < 1.1 CCR: 18.18 (8/44) 1.1 ⩽ AMH ⩽ 2.7 CCR: 0 (0/0) AMH > 2.7 CCR: 0 (0/0)	Not calculated
Kolanska *et al*.^20^ 218 patients	Percentage (absolute number)	All endometriosis CCR: 3 (5)	All endometriosis CCR: 6 (7)	All endometriosis 0.4
Ruggiero *et al*.^17^ 101 patients	Percentage	CCR: 16.3	CCR: 15.7	NS
Miscarriage rate				
Kolanska *et al*.^20^ 218 patients	Percentage (absolute number)	All endometriosis Fresh embryos MR < 12 GW: 6 (9/165) MR/ET: 7 (9/165) Freeze–thaw embryos MR < 12 GW: 2 (3/165) MR/ET: 7 (9/165) DE without either endometrioma or adenomyosis Fresh MR < 12 GW: 8 (2/25) MR/ET: 9 (2/23) Freeze-thaw MR < 12 GW: 0 (0/25) MR/ET: 0 (0/10) DE with endometrioma but without adenomyosis Fresh MR < 12 GW: 2 (1/50) MR/ET: 2 (1/45) Freeze–thaw MR < 12 GW: 2 (1/50) MR/ET: 6 (1/18) Endometrioma alone Fresh MR < 12 GW: 7 (1/14) MR/ET: 10 (1/10) Freeze–thaw MR < 12 GW: 7 (1/14) MR/ET: 33 (1/11) Endometriosis without adenomyosis Fresh MR < 12 GW: 6 (6/109) MR/ET: 6 (6/95) Freeze–thaw MR < 12 GW: 3 (3/109) MR/ET: 9 (3/35)	All endometriosis Fresh embryos MR < 12 GW: 3 (3/119) MR/ET: 4 (3/119) Freeze–thaw embryos MR < 12 GW: 1 (1/119) MR/ET: 3 (1/119) DE without either endometrioma or adenomyosis Fresh MR < 12 GW: 6 (1/16) MR/ET: 10 (1/10) Freeze–thaw MR < 12 GW: 0 (0/16) MR/ET: 0 (0/5) DE with endometrioma but without adenomyosis Fresh MR < 12 GW: 0 (0/36) MR/ET: 0 (0/29) Freeze–thaw MR < 12 GW: 0 (0/36) MR/ET: 0 (0/33) Endometrioma alone Fresh MR < 12 GW: 11 (1/9) MR/ET: 14 (1/7) Freeze–thaw MR < 12 GW: 0 (0/9) MR/ET: 0 (0/0) Endometriosis without adenomyosis Fresh MR < 12 GW: 2 (2/86) MR/ET: 3 (2/65) Freeze–thaw MR < 12 GW: 0 (0/86) MR/ET: 0 (0/26)	All endometriosis Fresh embryos MR < 12 GW: 0.4 MR/ET: 0.5 Freeze–thaw embryos MR < 12 GW: 0.9 MR/ET: 0.9 DE without either endometrioma or adenomyosis Fresh MR < 12 GW: 0.8337 MR/ET: 0.9047 Freeze–thaw MR < 12 GW: NA MR/ET: NA DE with endometrioma but without adenomyosis Fresh MR < 12 GW: NA MR/ET: 0.4189 Freeze–thaw MR < 12 GW: 0.3934 MR/ET: 0.3877 Endometrioma alone Fresh MR < 12 GW: 0.7417 MR/ET: 0.7872 Freeze–thaw MR < 12 GW: 0.4123 MR/ET: NA Endometriosis without adenomyosis Fresh MR < 12 GW: 0.2665 MR/ET: 0.3559 Freeze–thaw MR < 12 GW: 0.1210 MR/ET: 0.1258
Ruggiero *et al*.^17^ 101 patients	Percentage	4.8	6.3	NS
Pabuccu *et al*.^19^ 246 patients	Percentage	Stage I–II 2 Hx endometrioma without recurrence 2.4 Uni/bilateral endometrioma 3	Stage I–II 4 Hx endometrioma without recurrence 2.5 Uni/bilateral endometrioma 2.9	Stage I–II NS Hx endometrioma without recurrence NS Uni/bilateral endometrioma NS

All values shown as percentage (absolute number).

AMH, anti-Müllerian hormone; CCR, cycle cancellation rate; DE, deep endometriosis; ET, embryo transfer; Hx, history; MR, miscarriage rate; NS, not statistically significant.

## Discussion

### Main findings

Most studies found comparable clinical pregnancy and live birth rates between the long GnRH-agonist and GnRH-antagonist ovarian stimulation protocols. This is similar to that of women in the general IVF population and poor ovarian responders.^21–23^ In addition, fertilization rates were similar although the long GnRH-agonist protocol might be beneficial for some women with specific endometriosis subtypes and those with low ovarian reserve.

When comparing COS parameters, the GnRH-agonist protocol generally required greater gonadotrophin dose and longer treatment duration although this did not always reach significance. Adverse ART outcomes such as cycle cancellation rate and miscarriage rate were similar between the two protocols. The direct risk of developing OHSS could not be assessed because data regarding OHSS were not reported in these studies.

#### Interpreting pregnancy and LBRs

How pregnancy and LBRs are reported in studies is important to consider when discussing ART outcomes with patients. Since the number of embryos retrieved could be a predictive factor for pregnancy rate,^24^ excluding the patients who do not have a sufficient ovarian response by calculating the CPR per ET cycles^13,17,18^ would result in higher CPR as demonstrated by Kolanska *et al*.^20^ Future studies may consider reporting both CPR per cycle initiation and CPR per ET cycle as it provides better comparability between studies and more accuracy when discussing the chance of pregnancy at each stage of ART. Two multivariate regression analyses^13,18^ also identified the number of embryos and maternal age as predictive factors for IVF success which has been previously reported.^25^

#### Biological exploration

The precise mechanism by which GnRH analogues affect extra-pituitary reproductive tissues remains a topic of ongoing debate. Although most studies found no significant difference in CPR/LBR,^13,15–20^ Kolanska *et al.*^20^ were the only study to analyse both fresh and freeze–thaw cycles and found that the long GnRH-agonist protocol led to a significantly higher pregnancy rate in patients with endometriosis regardless of subtype. The authors suggested that this difference may be explained by the action of GnRH antagonists on the endometrium rather than ovaries which is in line with previous studies. In 2006, Ruan *et al.* using an IVF mice model compared GnRH-agonist *versus* GnRH-antagonist COS protocols and found that the expression of two uterine receptivity biomarkers (integrin β3 and leukaemia-inhibitory factor) during the implantation window was significantly lower in the GnRH-antagonist group. This correlated with a significantly lower implantation rate.^26^ A later case-control study in 2008 evaluated another receptivity marker, homeobox A10 (HOXA10) expression, from human endometrial biopsies and found decreased stromal and glandular cell HOXA10 expression in the GnRH-antagonist group.^27^ Although laboratory studies have found that the GnRH-antagonist protocol can reduce endometrial receptivity, in the clinic, pregnancy and birth rates in endometriosis patients are similar between both protocols^13–20^ and this is in agreement with meta-analyses that include poor and normal ovarian responders who have other causes of infertility.^9,21,22,28^

Laboratory studies have also found that the GnRH-antagonist protocol may lead to a poorer follicular microenvironment (higher nitric oxide concentration, increased superoxide dismutase expression and decreased insulin growth factor 1 and 2)^29,30^ which could impact oocyte yield. However, this is not necessarily reflected in human studies. Trials in women from other IVF populations comparing long GnRH-agonist protocols with GnRH-antagonist protocols report mixed findings on total and mature oocyte yield. In the general IVF population, the long GnRH-agonist may yield a higher number of oocytes, CPR and LBR.^9,13^ Meanwhile, in women with polycystic ovary syndrome who have a poor ovarian response, meta-analyses have shown that there is no significant difference in the total number of oocytes and mature oocytes retrieved.^9,22^ Our review found heterogeneous results among endometriosis patients similar to the results from meta-analyses on other infertile IVF populations. This demonstrates the variable effects of GnRH analogues on the ovaries.

While the well-known benefit of the GnRH-antagonist protocol is a reduced dosage of exogenous gonadotrophins required for ovarian stimulation, this may do more harm than good, especially in patients with a history of ovarian endometriomas. A comparative study by Al-Azemi *et al.*^31^ found that the presence of endometriomas significantly diminished ovarian reserve. Moreover, the surgical techniques during endometrioma cystectomy could damage the surrounding healthy ovarian tissue and vasculature increasing gonadotrophin resistance and negatively impact ovarian reserve.^32^ Hence, women with endometriomas usually require higher doses of recombinant FSH during ovarian stimulation due to a poorer ovarian response. Since the GnRH-agonist protocols are associated with higher gonadotrophin doses and longer COS duration, this may be advantageous for women with ongoing or resected endometriomas^15,19^ or with diminished ovarian reserve.

#### Fertilization rate as a marker for ART success

Fertilization rate is defined as the number of 2 pronuclear (2PN) oocytes that contain genetic information from both sperm and egg divided by the number of inseminated oocytes. Interestingly, fertilization rate has not been included in prior analyses comparing these two COS protocols. However, it can be a valuable parameter for women considering embryo cryopreservation since it can be a marker for cumulative pregnancy rate.^10,33^ Furthermore, higher fertilization rates can be an independent predictor for implantation rates. This is relevant when deciding the number of embryos to transfer, which is often problematic for IVF/ICSI providers.^34,35^ Although the fertilization rates of the two protocols were generally no different, this may be due to the selection of higher quality (mature) oocytes for fertilization. Therefore, the higher number of mature oocytes available for fertilization with the long GnRH-agonist protocol in conjunction with having a similar fertilization rate results in an increased cumulative pregnancy rate.

#### Selecting a GnRH analogue for ovarian stimulation

ART is an expensive treatment and the cost should be considered especially when ART is not subsidized or covered by insurance. A recent cost-effectiveness analysis comparing GnRH-agonist and GnRH-antagonist COS in the general IVF population by Jing *et al.* found that the GnRH-antagonist protocol is economically advantageous per fresh embryo cycles due to the shorter treatment duration, lower gonadotrophin dose required and lower incidence of OHSS. However, the cumulative ongoing pregnancy rate in both fresh and frozen embryo cycles is higher with the GnRH-agonist protocol due to the higher number of oocytes retrieved.^36^ Furthermore, the significantly shorter treatment duration with the GnRH-antagonist protocol would require fewer injections and lead to reduced treatment cost. Although most studies in our review found no significant differences in pregnancy or birth outcomes between the two protocols, the long GnRH-agonist protocol may still be favoured especially in patients with a history of ovarian endometriomas or diminished ovarian reserve. Thus, a patient-tailored approach should be sought, incorporating the patient’s disease characteristics and reproductive goals as a priority. As mentioned, the risk of developing OHSS could not be assessed due to the lack of available evidence and should be explored in future studies.

#### Strengths and limitations

To our knowledge, this is the first systematic review comparing ART outcomes following COS using the long GnRH-agonist protocol *versus* the GnRH-antagonist protocol specifically for women with endometriosis in accordance with PRISMA guidelines. Two authors (K.K.W.K. and S.O.) screened all the titles and completed bias/study quality assessment increasing the strength of our methodology. Several databases were searched without date restrictions minimizing the risk of selection bias. Authors were also sought for additional data and were provided by Drakopoulos *et al*.^14^

The single RCT and relatively small number of studies were the main limitations for this review. In addition, the small study numbers and heterogeneity of endometriosis stages/subtypes in each study did not allow for meta-analysis as one must consider the varying structural changes to the female reproductive anatomy. Due to the nature of observational studies, selection of endometriosis patients to either the GnRH-agonist or GnRH-antagonist protocol may be influenced by the clinician’s preferences. Fertility centres have also gained more experience with the GnRH-antagonist protocol throughout the years and an updated RCT would be preferred to minimize selection bias.

## Conclusion

This systematic review compared the long GnRH-agonist and GnRH-antagonist ovarian stimulation protocols and found similar CPRs and LBRs. However, the cumulative pregnancy rate may favour the long GnRH-agonist protocol due to the higher number of retrieved oocytes available for subsequent embryo cryopreservation. Women with ovarian endometriomas or poor ovarian reserve may benefit from the GnRH-agonist protocol due to greater gonadotrophin exposure resulting in an improved ovarian response. The GnRH-antagonist protocol is a sensible option for women with endometriosis, who want to lower the costs and duration of treatment. The risk of developing OHSS in endometriosis patients specifically could not be assessed and this outcome should be reported as a priority in future studies. A larger, well-powered RCT analysing patients according to endometriosis stage/subtype is needed. Ultimately, this review’s findings could help clinicians make an evidence-based decision when choosing a GnRH-analogue ovarian stimulation protocol while balancing treatment costs, stage/subtype of endometriosis and pregnancy goals of their patients.

## Supplemental Material

sj-docx-1-tae-10.1177_20420188231173325 – Supplemental material for Comparing ART outcomes in women with endometriosis after GnRH agonist versus GnRH antagonist ovarian stimulation: a systematic review Click here for additional data file. Supplemental material, sj-docx-1-tae-10.1177_20420188231173325 for Comparing ART outcomes in women with endometriosis after GnRH agonist versus GnRH antagonist ovarian stimulation: a systematic review by Kevin K.W. Kuan, Sean Omoseni and Javier A. Tello in Therapeutic Advances in Endocrinology and Metabolism

sj-docx-2-tae-10.1177_20420188231173325 – Supplemental material for Comparing ART outcomes in women with endometriosis after GnRH agonist versus GnRH antagonist ovarian stimulation: a systematic review Click here for additional data file. Supplemental material, sj-docx-2-tae-10.1177_20420188231173325 for Comparing ART outcomes in women with endometriosis after GnRH agonist versus GnRH antagonist ovarian stimulation: a systematic review by Kevin K.W. Kuan, Sean Omoseni and Javier A. Tello in Therapeutic Advances in Endocrinology and Metabolism

## Declarations

**Ethics approval and consent to participate:** Not applicable.

**Consent for publication:** Not applicable.

**Availability of data and materials:** The original contributions presented in the study are included in the article/Supplementary Tables, and further inquiries can be directed to the corresponding author.
