# Supplementary material for: Comparing ART outcomes in women with endometriosis after GnRH agonist versus GnRH antagonist ovarian stimulation: a systematic review
Source: Ther Adv Endocrinol Metab. 2023 Jul 4;14:20420188231173325. doi: 10.1177/20420188231173325 (PMC10331103; doi:10.1177/20420188231173325)
Supplement: sj-docx-1-tae-10.1177_20420188231173325 – Supplemental material for Comparing ART outcomes in women with endometriosis after GnRH agonist versus GnRH antagonist ovarian stimulation: a systematic review [file sj-docx-1-tae-10.1177_20420188231173325.docx]

**Supplementary Table 1.** Search strategy

**1.** **Ovid MEDLINE(R) ALL <1946 to June 10, 2022>**

1 exp Gonadotropin-Releasing Hormone/ 33625

2 exp Endometriosis/ 24258

3 1 and 2 1293

4 exp Infertility/ 71288

1. 3 and 4 192

**2. Embase <1974 to 2022 June 10>**

1 endometriosis/ 41700

2 agonist.mp. 297541

3 antagonist.mp. 462171

4 1 and 2 and 3 410

5 exp in vitro fertilization/ or infertility therapy/ or in vitro oocyte maturation/ or intracytoplasmic sperm injection/ or ovulation induction/ 98036

6 exp gonadorelin derivative/ 77092

7 4 and 6 334

8 gonadorelin agonist/ or gnrh analogue.mp. or gonadorelin antagonist/ 21268

9 4 and 8 322

1. 7 and 8 309

**3. Web of Science** (243 results)

All = (endometriosis or endometrioma)

All = (GnRH or “Gonadotropin-Releasing Hormone)

All = (infertility)
