# Supplementary material for: Comparing ART outcomes in women with endometriosis after GnRH agonist versus GnRH antagonist ovarian stimulation: a systematic review
Source: Ther Adv Endocrinol Metab. 2023 Jul 4;14:20420188231173325. doi: 10.1177/20420188231173325 (PMC10331103; doi:10.1177/20420188231173325)
Supplement: sj-docx-2-tae-10.1177_20420188231173325 – Supplemental material for Comparing ART outcomes in women with endometriosis after GnRH agonist versus GnRH antagonist ovarian stimulation: a systematic review [file sj-docx-2-tae-10.1177_20420188231173325.docx]

**Supplementary Table 3. Extracted Outcome Data Summary**

| **Study** | **Long GnRH-agonist** | **GnRH-antagonist** |
| --- | --- | --- |
| Zhao *et al.* (2020) | Gn dose (IU): 2594.24 ± 1057.56  Gn duration (d): 10.08 ± 2.22  #OR: 4.13 ± 2.04  FR (%): 78.46 ± 24.78  CP rate/ET cycle (%, n): 28.99 (20)  LBR (%, n): 24.64 (17)  IR (%, n): 17.16 (23)  Basal FSH (IU/L): 8.66 ± 2.50  EPR (%, n): 0 | Gn dose (IU): 2581.61 ± 827.11; p-value <0.001  Gn duration (d): 9.83 ± 1.74; p-value<0.001  #OR: 3.67 ± 1.92; p-value NS  FR (%): 73.52 ± 28.92; p-value NS  CP rate/ET cycle (%, n): 33.33 (29); p-value NS  LBR (%, n): 19.54 (17); p-value NS  IR (%, n): 18.01 (29); p-value NS  Basal FSH (IU/L): 9.30 ± 3.18; p-value = 0.644  EPR (%, n): 6.90 (2); p-value NS |
| Drakopoulos *et al.* (2018) | Stage I-II  Gn dose (IU): 2025  OSD (d) (median, IQR): 11 (9-12)  #OR (mean): 9 (6-13)  CP rate (%, n): 50 (21/42)  LBR (%, n): 42.8 (18)  FSH (IU/L), median (IQR): 6.3 (5-9)  #ET  (one embryo) (n,%): 20 (54)  (Two embryos) (n,%): 17(46)  Stage III-IV  Gn dose (IU, IQR): 2400 (2000-3000)  OSD (d, IQR): 11 (9-12)  #OR (mean, IQR): 8 (5-11)  CPR/patient(%, n/n): 34.3 (49/143)  LBR (%, n): 27.3 (39)  FSH (IU/L), median (IQR): 6.4 (5-8)  #ET:  (one embryo) (n,%): 92 (70.8)  (two embryos) (n,%): 38 (29.2) | Stage I-II  Gn dose (IU): 1650; p-value <0.001  OSD (d) (median, IQR): 9 (8-11); p-value = 0.1  #OR (mean, IQR): 7 (5-12); p-value = 0.09  CP rate (%, n): 36 (27/75); p-value = 0.14  LBR (%, n): 26.7 (20); p-value = 0.07  FSH (IU/L), median (IQR): 7.3(6-10); p-value = 0.33  #ET:  (one embryo) (n,%): 41 (60.3)  (two embryos) (n,%): 27(39.7)  Stage III-IV  Gn dose (IU, IQR): 2000 (1350-2625); p value< 0.001  OSD (d, IQR): 9 (8-11); p-value < .001  #OR (mean, IQR): 7 (5-11); p-value = 0.33  CPR/patient(%, n/n): 32.5 (41/126); p-value = 0.7  LBR (%, n): 23.8 (30); p-value = 0.5  FSH (IU/L), median (IQR): 7.3 (5-9); p-value = 0.07  #ET:  (one embryo) (n,%): 84 (73.8)  (two embryos) (n,%): 30(26.3) |
| Hosseini *et al.* (2017) | AMH <1.1  #OR: 3.04 ± 1.22  CP rate/ET (%, n): 5.5 (2/36)  Basal FSH (mIU/mL): 9.004  CCR: 13, 26.53%  1.1 ≤ AMH ≤ 2.7  #OR: 8.07 ± 3.36;  CP rate/ET (%, n): 41.3 (19/46)  Basal FSH (mIU/mL): 7.17  CCR: 0  AMH >2.7  #OR: 11.3 ± 3.02  CP rate/ET (%, n): 17.6 (6/34)  Basal FSH (mIU/mL): 8.17  CCR: 0 | AMH <1.1  #OR: 2.3 ± 1.72; p-value = 0.03  CP rate/ET (%, n): 13.6 (6/36); p-value = 0.2  Basal FSH (mIU/mL): 9.518; p-value = 0.319  CCR: 8, 18.18%  1.1 ≤ AMH ≤ 2.7  #OR: 6.8 ± 3.36; p-value = 0.08  CP rate/ET (%, n): 20.9 (9/43); p-value = 0.04  Basal FSH (mIU/mL): 7.68; p-value = 0.336  CCR: 0  AMH >2.7  #OR: 13.5 ± 3.6; p-value = 0.01  CP rate/ET (%, n): 39.4 (13/33); p-value = 0.06  Basal FSH (mIU/mL): 8.14; p-value = 0.94  CCR: 0 |
| Bastu *et al.* (2014) | Gn dose (IU): 3167.0 ± 1124.4  OSD (d): 11.00 ± 2.13  #MII OR: 7.93 ± 5.43  FR (%): 75.75 ± 32.98  CPR/patient (%, n): 20.5 (9/44)  Mean ET/cycle: 2.24± 1.11 | Gn dose (IU): 3261.1 ± 1653.9; p-value = NS  OSD (d): 10.16 ± 1.98; p-value = NS  #MII OR: 5.25 ± 5.51; p-value = 0.001  FR (%): 71.32 ± 32.94; p-value = NS  CPR/patient (%, n): 19.1 (8/42); p-value = NS  Mean ET/cycle: 1.98± 1.00 ; p-value = NS |
| Rodriguez-Purata *et al.* (2013) | Gn dose (IU): 2800 ± 1106  OSD (d): 10.5 ± 2.1  #OR: 11.2 ± 6.6  #MII OR: 8.3 ± 5.3  Basal FSH: 7.4±3.1  ET: 2.1±0.7  Pregnancy Rates/cycle  Group 1 = 41.9%  Group 2 = 39.7%  Group 3 = 15.4% | Gn dose (IU): 3105 ± 1395; p-value = 0.08  OSD (d): 10.7 ± 2.3; p-value = 0.006  #OR: 6.7 ± 4.4; p-value = 0.001  #MII OR: 5.3 ± 3.6; p-value = 0.001  Basal FSH: 9.2±3.8; p-value = 0.048  ET: 1.8±0.6; p-value = 0.001  Pregnancy Rates/cycle  Group 1 = 30%; p-value = 0.475  Group 2 = 36.4%; p-value = 0.77  Group 3 = 18.9%; p-value = 0.716 |
| Ruggiero *et al.* (2012) | rFSH dose (IU): 4817 ± 894  Basal FSH: 7.9±3.2  OSD (d): 11.8 ± 1.6  #OR: 3.8 ± 2.7  #MII OR:  3.3 ± 0.78  ET: 2±0.7  FR (%): 76.9;  CPR/ET (%): 16.7  CCR (%): 16.3  BCP(%): 10.7  EPR(%): 0  MR(%): 4.8 | rFSH dose (IU): 3923 ± 777; p-value= NS  Basal FSH : 7.1±4; p-value= 0.26 (NS)  OSD (d): 11.0 ± 1.7; p-value = 0.09 (NS)  #OR (m±SD): 4.8 ± 3; p-value= 0.15 (NS)  #MII OR: 4.1 ± 0.8; p-value= 0.21 (NS)  ET: 2.16±0.9; p-value= 0.28 (NS)  FR (%): 83.4; p-value = NS  CPR/ET (%): 19.3; p-value = NS  CCR(%): 15.7; p-value= NS  BCP(%): 12.5; p-value= NS  EPR(%): 1.9; p-value= NS  MR(%): 6.3; p-value= NS |
| Pabuccu *et al.* (2007) | Stage I-II  rFSH ampoules: 28.6 ± 8.7  COH duration (d): 10.1 ± 1.4  #OR: 13.3 ± 5.9  #MII OR: 9.6 ± 4.5  Basal FSH (IU/mL): 5.5 ± 1.1  FR (%): 76.4 ± 18.9  CPR/patient (%, n/n): 31.2 (15/48)  IR(%): 18.2  OSD: 10.1± 1.4  ET/cycle: 2.3± 0.5  AR (%): 2  Hx endometrioma without recurrence  rFSH ampoules (n): 32.1 ± 9.3  COH duration (d): 11.2 ± 1.5  #OR (n): 10.4 ± 5.9  #MII OR: 8.8 ± 4.6  FR (%): 71.2 ± 22.4  CP rate (%, n/n): 39 (16/41)  IR (%): 22.6  OSD: 11.2±1.5  ET/cycle: 2.1± 0.7  AR (%): 2.4  Uni/bilateral endometrioma  rFSH ampoules (#): 30.3 ± 8.7;  COH duration (d): 10.5 ± 1.6;  #OR: 8.2 ± 5.5  #MII OR: 6.5 ± 4.2  FR (%): 75.6 ± 15.4  CP rate (%, n): 24.2 (8/33)  IR (%): 14.8  OSD: 10.5±1.6  ET/cycle: 2.4± 0.8  AR (%): 3 | Stage I-II **(P-values all NS)**  rFSH ampoules (n): 27.4 ± 8.8;  COH duration (d): 9.9 ± 1.2  #OR: 12.1 ± 6.3  #MII OR (n): 8.9 ± 4.4  Basal FSH (IU/mL): 5.3 ± 0.9  FR (%): 73.7 ± 22.7  CPR/patient (%, n/n): 30 (15/50)  IR(%): 15.4  OSD: 9.9±1.2  ET/cycle: 2.4± 0.6  AR(%): 4  Hx endometrioma without recurrence (**NS if not stated)**  rFSH ampoules (n): 29.9 ± 8.5  COH duration (d): 10.5 ± 1.2  #OR (n): 8.3 ± 4.5  #MII OR: 4.3 ± 2.6; p-value = 0.0001  FR (%): 63.9 ± 21.1; p-value = 0.001  CP rate (%, n): 27.5 (11/40)  IR (%): 15.9  OSD: 10.5±1.2   ET/cycle: 2.1± 0.6  AR (%): 2.5  Uni/bilateral endometrioma (**NS if not stated)**  rFSH ampoules (#): 28.2 ± 8.7  COH duration (d): 9.9 ± 1.4  #OR: 6.7 ± 2.6; p-value = 0.002  #MII OR: 4.9 ± 1.6; p-value = 0.01  FR (%): 73.5 ± 23.7  CP rate (%, n): 20.5 (7/34)  IR (%): 12.5  OSD: 9.9± 1.4  ET/cycle: 2.5± 0.6  AR(%): 2.9 |
| Kolonska *et al.* (2017) | Total dose of FSH (UI) [median(range)]: 2425 (30-6600)  OSD (days) [median(range)]:11(6-92)  CCR (n,%): 5(3)  Fresh Embryos  PR per started cycle [n(%)]: 41 (25)  LBR per started cycle [n(%)] 31 (18)  Miscarriage< 12 GW [n(%)]: 9 (6)  PR per cycle with transfer [n(%)]:41 (29)  LBR per transfer [n(%)]: 31 (22)  Miscarriage per transfer [n(%)] 9 (7)  Freeze-thaw embryos  PR per started cycle [n(%)]: 8 (5)  LBR per started cycle [n(%)] 3 (2)  Miscarriage<12GW [n(%)] 3 (2)  PR per transfer [n(%)]:8 (16)  LBR per transfer [n(%)]: 3 (6)  Miscarriage per transfer [n(%)]: 9 (7)  Fresh+frozen embryos transfers  PR per started cycle [n (%)]: 48 (29)  LBR per started cycle [n(%)]: 34 (21)  PR per transfer [n(%]: 48 (29)  LBR per transfer [n(%)]: 34 (24)  **DIE without either endometrioma or adenomyosis**  Fresh  PR per started cycle [n (%)]: 7 (28)  LBR per started cycle [n(%)]: 5 (20)  Miscarriage<12GW [n(%)]: 2 (8)  PR per transfer [n(%]: 7 (30)  LBR per transfer [n(%)]: 5 (22)  Miscarriage per transfer [n(%)]: 2 (9)  Freeze-thaw  PR per started cycle [n(%)]: 0  LBR per started cycle [n(%)]: 0  Miscarriage<12 GW [n(%)]: 0  PR per transfer [n(%)]: 0  LBR per transfer [n(%)]: 0  Miscarriage per transfer [n(%)]: 0  **DIE with endometrioma but without adenomyosis**  Fresh  PR per started cycle [n (%)]: 14 (28)  LBR per started cycle [n(%)]: 9 (18)  Miscarriage<12GW [n(%)]: 1 (2)  PR per transfer [n(%]: 14 (31)  LBR per transfer [n(%)]: 9 (20)  Miscarriage per transfer [n(%)]: 1 (2)  Freeze-thaw  PR per started cycle [n(%)]: 2 (4)  LBR per started cycle [n(%)]: 1 (2)  Miscarriage<12 GW [n(%)]: 1 (2)  PR per transfer [n(%)]: 2 (11)  LBR per transfer [n(%)]: 1 (6)  Miscarriage per transfer [n(%)]: 1 (6)  **Endometrioma alone**  Fresh  PR per started cycle [n(%)]: 1 (7)  LBR per started cycle [n(%)]: 0  Miscarriage<12GW [n(%)]: 1 (7)  PR per transfer [n(%]: 1 (10)  LBR per transfer [n(%)]: 0  Miscarriage per transfer [n(%)]: 1 (10)  Freeze-thaw  PR per started cycle [n(%)]: 2 (14)  LBR per started cycle [n(%)]: 1 (7)  Miscarriage<12 GW [n(%)]: 1 (7)  PR per transfer [n(%)]: 2 (67)  LBR per transfer [n(%)]: 1 (33)  Miscarriage per transfer [n(%)]: 1 (33)  **Endometriosis without adenomyosis**  Fresh  PR per started cycle [n (%)]: 27 (25)  LBR per started cycle [n(%)]: 16 (15)  Miscarriage<12GW [n(%)]: 6 (6)  PR per transfer [n(%]: 27 (28)  LBR per transfer [n(%)]: 16 (17)  Miscarriage per transfer [n(%)]: 6 (6)  Freeze-thaw  PR per started cycle [n(%)]: 5 (5)  LBR per started cycle [n(%)]: 2 (2)  Miscarriage<12 GW [n(%)]: 3 (3)  PR per transfer [n(%)]: 5 (14)  LBR per transfer [n(%)]: 2 (6)  Miscarriage per transfer [n(%)]: 3 (9) | Total dose of FSH (UI) [median(range)]: 2500 (14-5850); p-value = 0.4  OSD (days) [median(range)]:11(6-18); p-value = 0.3  CCR (n,%): 7(6); p-value =0.4  Fresh Embryos  PR per started cycle [n(%)]: 15 (13); p-value = 0.017  LBR per started cycle [n(%)]: 9 (8); p-value = 0.04  Miscarriage< 12 GW [n(%)]: 3 (3)  PR per cycle with transfer [n(%)]: 15 (17); p-value = 0.053  LBR per transfer [n(%)]: 9 (10); p-value = 0.02  Miscarriage per transfer [n(%)]: 3 (4); p-value = 0.5  Freeze-thaw embryos  PR per started cycle [n(%)]: 8 (7); p-value = 0.7  LBR per started cycle [n(%)]: 8 (7); p-value = 0.09  Miscarriage<12 GW [n(%)]: 1 (1); p-value = 0.9  PR per transfer [n(%)]: 8 (22); p-value = 0.7  LBR per transfer [n(%)]: 8 (22); p-value =0.001  Miscarriage per transfer [n(%)]: 1(3); p-value = 0.9  Fresh+frozen embryos transfers  PR per started cycle [n (%)]: 22 (18); p-value = 0.06  LBR per started cycle [n(%)]: 17 (14); p-value = 0.19  PR per transfer [n(%]: 22 (18); p-value = 0.1  LBR per transfer [n(%)]: 17 (18); p-value = 0.29  **DIE without either endometrioma or adenomyosis**  Fresh  PR per started cycle [n (%)]: 1 (6); p-value = 0.0865  LBR per started cycle [n(%)]: 0; p-value = 0.0563  Miscarriage<12GW [n(%)]: 1 (6); p-value = 0.8337  PR per transfer [n(%]: 1 (10); p-value = 0.2081  LBR per transfer [n(%)]: 0; p-value = 0.1095  Miscarriage per transfer [n(%)]: 1 (10); p-value = 0.9047  Freeze-thaw  PR per started cycle [n(%)]: 1 (6); p-value = 0.2057  LBR per started cycle [n(%)]: 1 (6); p-value = 0.2057  Miscarriage<12 GW [n(%)]: 0  PR per transfer[n(%)]: 1 (20); p-value = 0.1432  LBR per transfer[n(%)]: 1 (20); p-value = 0.1432  Miscarriage per transfer [n(%)]: 0  **DIE with endometrioma but without adenomyosis**  Fresh  PR per started cycle [n (%)]: 5 (14); p-value = 0.1197  LBR per started cycle [n(%)]: 2 (6); p-value = 0.0883  Miscarriage<12GW [n(%)]: 0  PR per transfer [n(%]: 5 (17); p-value = 0.1824  LBR per transfer [n(%)]: 2 (7); p-value = 0.1219  Miscarriage per transfer [n(%)]: 0; p-value = 0.4189  Freeze-thaw  PR per started cycle [n(%)]: 3 (8); p-value = 0.3969  LBR per started cycle [n(%)]: 0; p-value = 0.3934  Miscarriage<12 GW [n(%)]: 0; p-value = 0.3934  PR per transfer [n(%)]: 3 (23); p-value = 0.3714  LBR per transfer [n(%)]: 0; p-value = 0.3877  Miscarriage per transfer [n(%)]: 0; p-value = 0.3877  **Endometrioma alone**  Fresh  PR per started cycle [n (%)]: 2 (22); p-value = 0.2946  LBR per started cycle [n(%)]: 1 (11); p-value = 0.2022  Miscarriage<12GW [n(%)]: 2 (8); p-value = 0.7417  PR per transfer [n(%]: 2 (29); p-value = 0.3229  LBR per transfer [n(%)]: 1 (14); p-value = 0.2179  Miscarriage per transfer [n(%)]: 1 (14); p-value = 0.7872  Freeze-thaw  PR per started cycle [n(%)]: 0; p-value = 0.2354  LBR per started cycle [n(%)]: 0; p-value = 0.4123  Miscarriage<12 GW [n(%)]: 0; p-value = 0.4123  PR per transfer [n(%)]: 0  LBR per transfer [n(%)]: 0  Miscarriage per transfer [n(%)]: 0  **Endometriosis without adenomyosis**  Fresh  PR per started cycle [n (%)]: 10 (12); p-value = 0.0201  LBR per started cycle [n(%)]: 4 (5); p-value = 0.0219  Miscarriage<12GW [n(%)]: 2 (2); p-value = 0.2665  PR per transfer [n(%]: 10 (15); p-value = 0.0548  LBR per transfer [n(%)]: 4 (6); p-value =0.4470  Miscarriage per transfer [n(%)]: 2 (3); p-value = 0.3559  Freeze-thaw  PR per started cycle [n(%)]: 6 (7); p-value = 0.4727  LBR per started cycle[n(%)]: 1( 1); p-value = 0.7050  Miscarriage<12 GW [n(%)]: 0; p-value = 0.1210  PR per transfer [n(%)]: 6 (23); p-value = 0.3771  LBR per transfer [n(%)]: 1 (4); p-value = 0.7386  Miscarriage per transfer [n(%)]: 0; p-value = 0.1258 |

All values in mean ± standard deviation unless otherwise stated

Abbreviations (alphabetical order): #: number. CCR: cycle cancellation rate. COH: controlled ovarian hyperstimulation. CP: clinical pregnancy. d: days. END: early neonatal death. EPR: ectopic pregnancy rate. ET: embryo transfer. FR: fertilization rate. FSH: follicle stimulating hormone. GnRH-a: Gonadotrophin-releasing hormone agonist. Gn dose: gonadotrophin dose. GnRH-ant: gonadotrophin-releasing hormone antagonist. Hx: history. IU: units. IVF: *in-vitro* fertilization. LBR: live birth rate. MII OR: metaphase 2 (mature) oocytes retrieved. N=: number of patients. NS=: not statistically significant. OR: oocytes retrieved. NS: Not significant. OSD: ovarian Stimulation duration. rFSH: recombinant follicle stimulating hormone. SD: standard deviation.
